# An “interlocking” core-shell architecture stabilises perovskite nanocrystal emitters

**DOI:** 10.1038/s41377-026-02441-z

**Published:** 2026-09-08

**Authors:** Xinyu Shen, Henry J. Snaith

**Affiliations:** Clarendon Laboratory, Department of Physics, University of Oxford, Oxford, OX1 3PU UK

**Keywords:** Optics and photonics, Electronics, photonics and device physics

## Abstract

The practical application of perovskite nanocrystals has been hindered by their intrinsic instability, arising from the coupled effects of soft ionic lattices, ion migration, and surface reactions. A hierarchical shell strategy now addresses these intertwined degradation pathways through lattice-interface “interlocking”, achieving *T*_90_ values exceeding 27,000 h under continuous blue-light exposure. Notably, the resulting hierarchical shell-perovskite nanocrystals enable reliable colour-conversion display prototypes.

## Abstract (web summary)

Hierarchical shell enables lattice-interface interlocking, suppresses coupled degradation, delivers near-unity photoluminescence quantum yields and *T*_*90*_ exceeding 27,000 hours under continuous blue-light exposure, and demonstrates efficient perovskite nanocrystal color-conversion display prototypes.

## Graphical abstract

**Figure:**
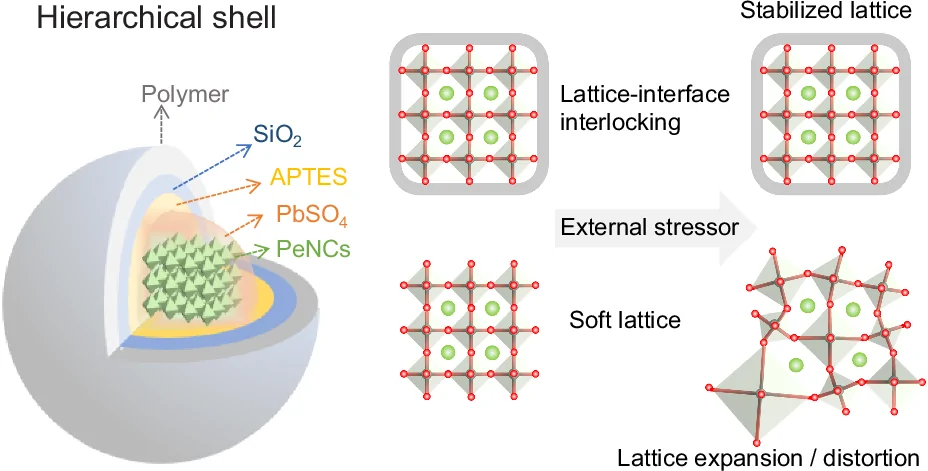

In the modern information era, human-machine interaction is undergoing a profound transformation, making display one of the most important optoelectronic technologies^1^. The development of next-generation displays places increasing demands on light-emitting materials with high colour purity, high efficiency, and wide colour gamut to ensure faithful information delivery^2,3^. Compared with already-commercilised emitters such as phosphors, organic semiconductors, and quantum dots, metal halide perovskite nanocrystals have attracted particular attention because of their high photoluminescence quantum yields (PLQY), narrow emission linewidths and extremely strong absorption strengths, which enable excellent colour purity, efficient “photon recycling”, nearly full coverage of the Rec. 2020 colour gamut, and usefulness in “in-pixle” colour conversion, required for microdisplays^4–6^. Despite these advantages, their practical application remains constrained by intrinsic instability.

Metal halide perovskite nanocrystals are nanoscale ionic semiconductors with an ABX_3_ chemical formula, consisting of monovalent organic or inorganic cations, divalent metal cations, and halide anions^7^. Their ionic nature, together with relatively low formation energies and low ion-migration barriers, facilitates their synthesis and enables versatile surface chemistry^8^. However, these same features also underlie their instability: surface ligands can readily detach, while moisture, oxygen, light, and heat can all induce structural and chemical degradation^9^.

Over the past decade, multiple strategies have been explored to mitigate these intrinsic limitations. Composition engineering, including A-site multi-cation alloying, B-site divalent-cation doping, and X-site pseudohalide substitution, has been widely used to stabilize the perovskite lattice^10–12^. Meanwhile, the surface of nanocrystals is known to critically influence both luminescence efficiency and long-term stability^13^. CdSe and InP nanocrystals reached commercial viability by employing covalently bonded protective shells of ZnS/Se^3^. Surface-management approaches for perovskite nanocrystals, such as core-shell engineering, polymer matrix encapsulation, oxide coating, and crosslinked surface ligands, have been developed to resist degradation triggered by external stressors^14–17^. However, the perovskite nanocrystals still struggle to maintain the high PLQYs over long-term stressing, required for commercially viable operational lifetimes. More importantly, lattice instability and surface degradation have often been treated separately, whereas their coupled nature remains insufficiently addressed. This prompts a rethinking of how provskite nanocrystal stability should be engineered.

These considerations make the work of Zeng and co-workers particularly noteworthy^18^. They developed a hierarchical shell composed of interbonded lead sulfate (PbSO_4_)-silicon dioxide (SiO_2_)-siloxane polymer layers (Fig. 1a). The key to the success of this strategy lies in the rational design of the molecule, 3-aminopropyltriethoxysilane sulfate ((APTES)_2_SO_4_), which both forms and serves as a bridge between the inner PbSO_4_ layer and the outer siloxane polymer layer. The SO_4_^2−^ binds with Pb^2+^ to form the inner PbSO_4_ layer, which directly anchors the perovskite lattice, effectively locks surface ions, and passivates surface trap states. APTES further enables the perovskite nanocrystal-PbSO_4_ structures to form a SiO_2_ shell with uniform thickness while maintaining their original structure. This effectively suppresses agglomeration and avoids the formation of broad size distributions of nanocrystals, particularly in undesirable configurations where multiple nanocrystal cores become unintentionally embedded within a single shared shell^19^.

**Fig. 1:**
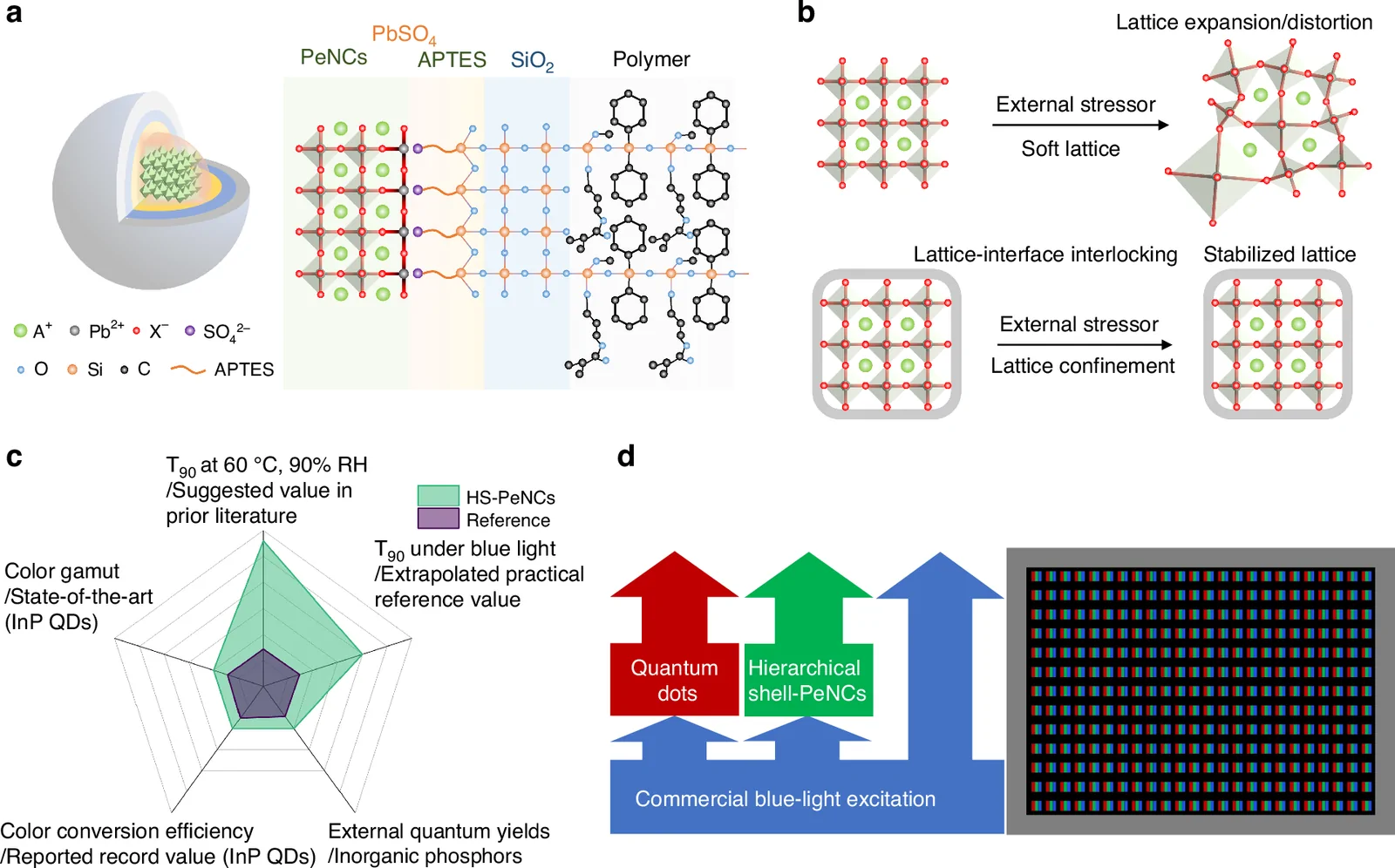
Hierarchical shell enabled lattice-interface interlocking in perovskite nanocrystal emitters. **a** Schematic illustration of the hierarchical shell-perovskite nanocrystal structure. **b** Schematic illustration of lattice-interface interlocking in hierarchical shell–perovskite nanocrystals. **c** Comparison of key performance metrics with representative reference systems. **d** Schematic illustration of the colour-conversion display prototype

Notably, they revealed a lattice-interface interlinked degradation pathway in colloidal perovskite nanocrystals through systematic experiments and theoretical calculations. The well-defined and stabilized core-shell structures enabled by the hierarchical design provide a robust platform for disentangling the coupled effects of lattice dynamics and interfacial chemistry, which are otherwise difficult to isolate. Lattice strain triggered by external stressors is effectively suppressed through their so-called lattice-interface interlocking (Fig. 1b).

As shown in Fig. 1c, the hierarchical shell-MAPbBr_3_ films retain close to 100% PLQY, with an external quantum yield of 91.4%, a record colour-conversion efficiency (defined as the ratio of converted light intensity to absorbed light intensity^20^) of 69.6%, and a 137% National Television Standards Committee (NTSC) colour gamut. These values substantially exceed representative benchmarks, including external quantum yields of inorganic phosphors (∼65%)^21^, colour conversion efficiency of InP quantum dots (∼52%)^22^, and NTSC gamut of InP quantum dots (∼94%).

Meanwhile, hierarchical shell-MAPbBr_3_ exhibits exceptional stability with *T*_90_ = 3900 h at 60 °C and 90% relative humidity in the dark, and >27,000 h under continuous blue-light exposure of 180 W/m^2^ at room temperature. The former is comparable to commercial targets summarized in prior literature^9^, whereas the authors claim that the stability under prolonged light exposure surpasses industrial benchmarks. We do note, however, that it is not straightforward to estimate the actual incident blue-light irradiance and steady-state operating temperatures required for realistic operating lifetimes in different device applications. Therefore, although these results are extremely encouraging, a more detailed assessment of suitable use in different targeted applications is required before it can be robustly claimed that the stability surpasses industrial requirements.

In addition, the hierarchical shell strategy is demonstrated to be compositionally general and scalable, enabling stable emission across 409–783 nm, including mixed-halide and hybrid perovskite compositions. In mixed-halide systems, the photostability is improved owing to the suppression of ion migration and halide-phase separation.

Beyond photophysical properties, the hierarchical shell structure also enhances practical processability by preventing lead leakage and enabling compatibility with scalable fabrication techniques, including roll-to-roll coating, inkjet printing, and photolithographic patterning with the aqueous developer AZ 300 MIF at pixel densities exceeding 3500 pixels per inch. Furthermore, hierarchical shell-MAPbBr_3_ has been demonstrated in a colour-conversion display prototype (Fig. 1d), achieving 4K resolution and nearly covering the Rec.2020 color gamut. Together, these attributes highlight the strong potential of this approach for high-resolution display technologies and represent an important step toward practical applications.

More broadly, this work exemplifies the importance of a mechanism-driven approach to materials design. By identifying the coupled origin of instability and addressing it through rational structural engineering, it provides a pathway to bridge intrinsic material limitations with practical performance requirements. Rather than overturning existing understanding, it refines it by integrating lattice dynamics and interfacial chemistry into a unified framework for stabilizing perovskite nanocrystals. As such, it offers a broadly applicable design principle for improving stability across a wide range of perovskite optoelectronic applications.
